# ASL/ALT Ratio in Familial and Sporadic Parkinson's Disease: Insights From Cross‐Sectional Logistic Analysis

**DOI:** 10.1002/brb3.71416

**Published:** 2026-04-22

**Authors:** Wen Zhou, Tianfang Zeng, Duan Liu, Ruijuan Pang

**Affiliations:** ^1^ West China School of Medicine Sichuan University, Sichuan University Affiliated Chengdu Second People's Hospital, Chengdu Second People's Hospital Chengdu Sichuan China

**Keywords:** familial Parkinson's disease (fPD), Parkinson's Progression Markers Initiative (PPMI), retrospective cross‐sectional study, sporadic Parkinson's disease (sPD), the aspartate aminotransferase (AST) to alanine aminotransferase (ALT) ratio (AST/ALT)

## Abstract

**Background**: Parkinson's disease (PD) exhibits genetic heterogeneity with varying clinical presentations. The aspartate aminotransferase (AST) to alanine aminotransferase (ALT) ratio has emerged as a potential indicator of neurodegenerative processes, yet its relationship with PD genetic subtypes remains unexplored.

**Objective**: This study aims to investigate the relationship between the AST to ALT ratio (AST/ALT) and different genetic subtypes of PD.

**Methods**: This retrospective analysis included 1315 PD patients (1005 sporadic, 310 familial) from Parkinson's Progression Markers Initiative (PPMI) databases. Multivariable logistic regression models, restricted cubic splines, and inflection point analysis were employed to assess the relationship between the AST/ALT ratio and genetic subtypes of PD, adjusting for demographic, clinical, and biochemical covariates. Subgroup and sensitivity analyses were conducted to evaluate robustness, and a predictive model was developed using stepwise selection.

**Results**: Compared with sporadic PD (adjusted odds ratio [OR] 1.69, 95% confidence interval 1.19–2.39, *p* = 0.003). A nonlinear relationship was identified (nonlinearity *p* = 0.002), with an inflection point at 1.503. Above this threshold, the association became markedly stronger (OR 22.27, *p* = 0.0007), whereas no significant association was detected below it. Findings remained consistent across all subgroup and sensitivity analyses. A predictive model incorporating the AST/ALT ratio, along with clinical and demographic factors, achieved a moderate discriminatory ability (area under the curve 0.758).

**Conclusions**: The AST/ALT ratio demonstrates a significant, nonlinear association with genetic subtypes of PD, particularly among individuals with higher ratio values. This readily accessible serum marker may hold promise for refining PD subtyping and informing personalized management strategies, warranting validation in prospective and diverse cohorts.

## Graphical abstract

This study reveals a nonlinear association between the AST/ALT ratio and familial versus sporadic Parkinson's disease. Above a threshold of 1.503, the ratio is strongly associated with familial PD, suggesting this simple serum marker may help distinguish genetic subtypes.

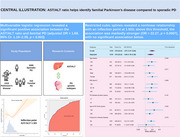

## 1 Introduction

According to the Global Burden of Disease Study, Parkinson's disease (PD) is the fastest‐growing neurological disorder, with significant increases observed in age‐standardized prevalence, disability, and mortality rates (GBD 2015 Neurological Disorders Collaborator Group 2017). This finding underscores the importance of PD as a global health issue and highlights the need for enhanced understanding, diagnosis, and treatment of this disease. PD is characterized by significant clinical heterogeneity, evident in aspects such as age of onset, clinical presentation, disease progression, and treatment response. Distinct PD subtypes exhibit not only varying clinical presentations and prognoses but also diverse underlying pathophysiological mechanisms (Simon et al. 2020). This heterogeneity suggests that subtype‐specific, personalized therapeutic approaches may be more effective. Although the majority of PD cases are sporadic, it is widely accepted that the interplay between genetic susceptibility and environmental factors plays a crucial role in disease development (Sulzer 2007). Moreover, subtype‐specific therapies for monogenic PD have shown promise in clinical trials (Tolosa et al. 2020; Poewe et al. 2020). Therefore, an in‐depth investigation into the genetic characteristics of different PD subtypes is essential. It will not only elucidate the pathophysiological mechanisms of each subtype but also provide a theoretical basis for the development of precision medicine strategies, which is of great significance for improving the prognosis of PD patients.

The aspartate aminotransferase (AST) to alanine aminotransferase (ALT) ratio (AST/ALT) is a well‐established marker commonly associated with liver injury. However, recent studies have unveiled its potential relevance to neurodegenerative diseases. For instance, in Alzheimer's disease, elevated liver enzyme levels have been correlated with cognitive impairment, with higher AST/ALT ratios associated with an increased risk of cognitive deficits (Li et al. 2022). Moreover, higher AST/ALT ratios have been reported in AD cohorts compared to controls (Nho et al. 2019; Zholdasbekova et al. 2024). Additionally, one recent study has found an association between elevated AST/ALT ratios and an increased risk of PD (Gao et al. 2025). These findings suggest that the AST/ALT ratio may serve as a potential biomarker for neurodegenerative processes.

Parkinson's Progression Marker Initiative (PPMI) database integrates whole‐genome sequencing data, biochemical blood markers (including AST/ALT), multimodal neuroimaging, and longitudinal clinical assessments from multicenter PD patients globally (Marek et al. 2011), providing a unique resource for elucidating genetic subtype‐specific biomarkers. Our study utilizes data from the PPMI database to explore the association between the AST/ALT ratio and different genetic subtypes of PD. This study seeks to elucidate whether the AST/ALT ratio can provide insights into the genetic heterogeneity of PD, potentially offering a novel avenue for subtype‐specific therapeutic strategies.

## 2 Methods and Materials

### 2.1 Data Sources and Study Population

**FIGURE 1:**
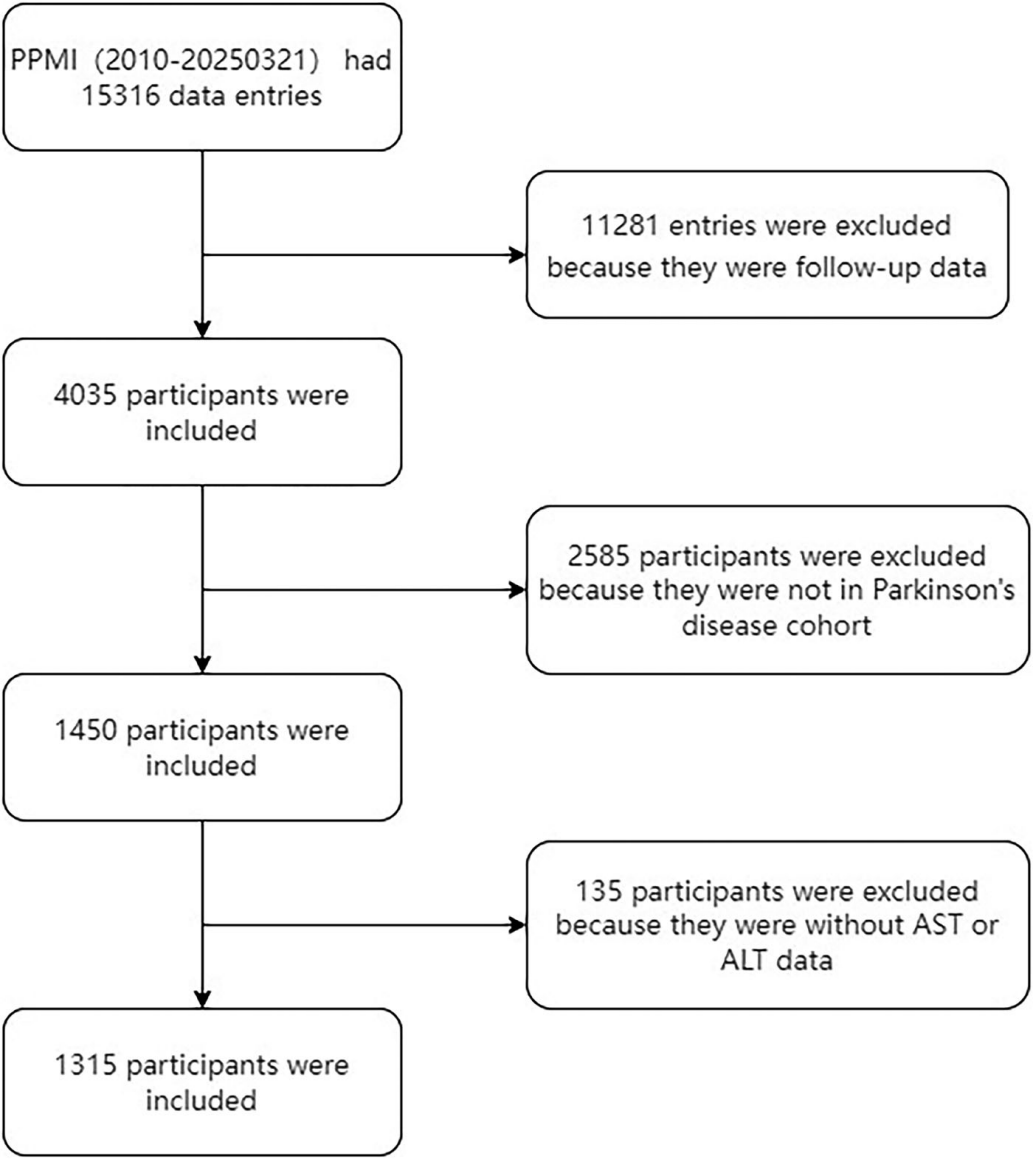
Flowchart of the study cohort. ALT, alanine aminotransferase; AST, aspartate aminotransferase; PPMI, Parkinson's Progression Markers Initiative.

The study utilized data acquired from Parkinson's Progression Markers Initiative (PPMI; http://www.ppmi‐info.org) accessed on March 21, 2025, as previously described (Zhou et al. 2025). Its primary objective is to identify clinical and biological markers associated with the heterogeneity and progression of PD. This study included patients with a confirmed PD diagnosis, initially determined by movement disorder specialists and site investigators and subsequently validated by a central consensus committee (Figure 1). The PPMI study obtained ethical approval from the Institutional Review Boards of all participating institutions, and documented informed consent was secured from each enrolled individual. Baseline assessments were conducted at the time of enrollment, and all variables were derived from these initial evaluations. The racial composition of our study population was predominantly White (*n* = 1236; 94.5%), followed by Asian (*n* = 17; 1.3%), Black (*n* = 13; 1.0%), and other races (*n* = 42; 3.2%). Importantly, this study utilized the publicly available PPMI dataset, from which all data are de‐identified; accordingly, no additional IRB approval was required for our analysis.

### 2.2 Assessment Tools

This study employed the movement disorder society‐unified Parkinson's disease rating scale (MDS‐UPDRS), a validated instrument that offers comprehensive assessment of both motor and non‐motor symptoms in PD. As an updated version of the original UPDRS, it was selected for its proven reliability and sensitivity in capturing motor symptom severity and progression.

Non‐motor symptoms were assessed using the Epworth Sleepiness Scale (ESS), REM Sleep Behavior Disorder Screening Questionnaire (RBDSQ), 15‐item Geriatric Depression Scale (GDS), and the Montreal Cognitive Assessment (MOCA). Dopamine transporter imaging utilizing DaTscan was carried out at predefined PPMI imaging facilities in strict adherence to the standardized protocol accessible via the PPMI website. All enrolled individuals underwent mandatory DaTscan procedures to quantify dopamine transporter binding capacity, with analysis protocols rigorously aligned to the PPMI Imaging Technical Operations Manual.

Laboratory assessments of hematological and biochemical parameters were uniformly executed at Covance laboratories as stipulated in the study design. Evaluated markers encompassed serum analytes including total protein, total bilirubin, serum uric acid, creatinine, albumin, serum glucose, AST, and ALT. The AST/ALT ratio was computed using the formula: AST (U/L) divided by ALT (U/L).

### 2.3 Statistical Methods

Descriptive statistics were used to summarize baseline characteristics. Continuous variables with normal distribution were presented as mean ± standard deviation, whereas those with non‐normal distribution were reported as median (interquartile range, IQR). Categorical variables were expressed as frequencies and percentages.

To assess the independent association between the AST/ALT ratio and PD genetic subtypes, multivariable logistic regression models were constructed using multiply imputed datasets. Covariates included age, sex, body mass index (BMI), race, disease duration, UPDRS total score, serum glucose, serum uric acid, albumin, total bilirubin, creatinine, antihypertensives, antidiabetics, lipid‐lowering drug, and nonsteroidal anti‐inflammatory drugs (NSAIDs). The selection of these confounders was based on clinical relevance, existing scientific literature, and their known associations with the outcomes of interest, including cases where they led to a change in the effect estimate exceeding 10%. The odds ratios (ORs) and 95% confidence intervals (CIs) were reported for each genetic subtype.

To explore the potential nonlinear relationship between the AST/ALT ratio and PD genetic subtypes, restricted cubic splines with three knots were implemented. The inflection point was identified using the method of piecewise linear regression, where the AST/ALT ratio was modeled as a continuous variable.

Subgroup analyses were performed to evaluate the relationship between the AST/ALT ratio and PD genetic subtypes within different demographic and clinical strata, including age groups (<65 years vs. ≥65 years), sex (male vs. female), BMI (<25, 25–30, ≥30 kg/m^2^), disease duration (<3 years vs. ≥3 years), age at onset (<50 years vs. ≥50 years), and the presence of any endocrine or muscle disease (yes vs. no).

To verify the robustness of our results, several sensitivity analyses were conducted. First, participants with missing AST/ALT ratio data were excluded, and the analyses were re‐run to ensure that missing data did not bias the results. Second, participants with AST or ALT levels exceeding the upper limit of normal were excluded to control for potential liver function abnormalities. Third, given that muscle and endocrine disorders can influence aminotransferase levels, we performed an additional sensitivity analysis excluding all participants diagnosed with any such condition to assess whether our primary findings were driven by this subgroup.

To evaluate the sensitivity and specificity of the AST/ALT ratio in differentiating familial PD from sporadic PD, we performed a receiver operating characteristic (ROC) analysis. In the model‐building process, we considered the following potential confounding variables: age, sex, disease duration, BMI, race, UPDRS total score, serum glucose, serum uric acid, albumin, total bilirubin, creatinine, antihypertensives, antidiabetics, lipid‐lowering drug, NSAIDs, and the AST/ALT ratio. To identify the most significant predictors, we employed a stepwise selection method based on the adjusted *R*‐squared value. The variables retained in the final model were then used to construct a predictive model to assess their combined ability to discriminate between familial and sporadic PD. Using the selected variables, we constructed a predictive model and calculated the area under the ROC curve (AUC) to evaluate the model's discriminatory power.

Statistical computations were implemented through R 4.2.2 (R Foundation) and Free Statistics v1.9, with hypothesis testing employing a two‐sided significance threshold of 0.05.

## 3 Result

### 3.1 Baseline Characteristics of Participants

A total of 1315 participants were included in the study, comprising 1005 individuals with sporadic PD and 310 with familial PD. Baseline characteristics are summarized in Table 1. Overall, 817 participants (62.1%) were male, with a higher proportion of males in the sporadic PD group (65.2%) compared to the familial PD group (52.3%). The mean age of the entire cohort was 62.9 years, with the sporadic PD group having a slightly higher mean age of 63.5 years compared to 60.9 years in the familial PD group.

**TABLE 1:** Clinical characteristics of the study population by Parkinson's disease (PD) subtype.

		PD		
Variable	Total (*n* = 1315)	Sporadic PD (*n* = 1005)	Familial PD (*n* = 310)	*p* value
Age (years), mean ± SD	62.9 ± 9.7	63.5 ± 9.3	60.9 ± 10.7	<0.001
Sex, *n* (%)				<0.001
Female	498 (37.9)	350 (34.8)	148 (47.7)	
Male	817 (62.1)	655 (65.2)	162 (52.3)	
Education (years), mean ± SD	15.8 ± 3.1	15.9 ± 2.8	15.2 ± 3.9	<0.001
Race, *n* (%)				0.133
White	1236 (94.5)	940 (94.2)	296 (95.5)	
Black	13 (1.0)	12 (1.2)	1 (0.3)	
Asian	17 (1.3)	16 (1.6)	1 (0.3)	
Other	42 (3.2)	30 (3)	12 (3.9)	
BMI (kg/m^2^), median (IQR)	26.9 ± 4.9	27.0 ± 5.0	26.8 ± 4.6	0.493
Age onset, mean ± SD	59.9 ± 10.3	61.1 ± 9.5	56.1 ± 11.6	<0.001
Onset years, median (IQR)	2.1 (1.2, 3.4)	1.9 (1.1, 2.9)	3.5 (2.0, 6.0)	<0.001
Total protein (g/L), mean ± SD	69.8 ± 4.1	69.5 ± 4.0	70.7 ± 4.2	<0.001
Total bilirubin (umol/L), mean ± SD	9.9 ± 5.6	10.1 ± 5.7	9.3 ± 5.5	0.05
Serum uric acid (umol/L), mean ± SD	303.8 ± 76.6	307.4 ± 75.3	292.1 ± 79.6	0.002
Creatinine (umol/L), mean ± SD	82.0 ± 17.9	82.8 ± 17.9	79.5 ± 18.0	0.004
Albumin (g/L), mean ± SD	44.0 ± 3.5	44.0 ± 3.6	43.8 ± 3.1	0.404
Serum glucose (mmol/L), mean ± SD	5.6 ± 1.2	5.6 ± 1.2	5.5 ± 1.1	0.253
AST (U/L), mean ± SD	22.1 ± 9.2	22.3 ± 9.8	21.3 ± 7.0	0.092
ALT (U/L), mean ± SD	21.7 ± 12.6	22.1 ± 12.8	20.5 ± 11.8	0.053
De‐Ritis ratio, mean ± SD	1.1 ± 0.4	1.1 ± 0.3	1.2 ± 0.6	<0.001
MoCA mean ± SD	26.8 ± 2.6	27.0 ± 2.4	26.2 ± 3.2	<0.001
ESS, median (IQR)	5.0 (3.0, 8.0)	5.0 (3.0, 8.0)	5.0 (3.0, 9.0)	0.116
RBD, median (IQR)	3.0 (2.0, 6.0)	3.0 (2.0, 6.0)	4.0 (2.0, 6.0)	0.114
GDS, median (IQR)	2.0 (0.0, 3.0)	2.0 (0.0, 3.0)	2.0 (1.0, 5.0)	<0.001
UPDRS 1 score, median (IQR)	6.0 (3.0, 9.0)	5.0 (3.0, 9.0)	7.0 (3.8, 12.0)	<0.001
UPDRS 2 score, median (IQR)	5.5 (3.0, 9.0)	5.0 (3.0, 9.0)	6.0 (3.0, 10.0)	0.029
UPDRS 3 score, mean ± SD	22.5 ± 10.1	22.4 ± 9.6	22.8 ± 11.6	0.599
UPDRS total score, mean ± SD	35.8 ± 15.5	35.2 ± 14.6	38.3 ± 18.4	0.004
Caudate measurement, mean ± SD	2.0 ± 0.6	2.0 ± 0.6	1.8 ± 0.6	<0.001
Putamen measurement, mean ± SD	0.9 ± 0.3	0.9 ± 0.3	0.8 ± 0.3	<0.001
Striatum measurement, mean ± SD	1.4 ± 0.4	1.4 ± 0.4	1.3 ± 0.4	<0.001
Endocrine or muscle disease, *n* (%)				0.155
No	1051 (79.9)	812 (80.8)	239 (77.1)	
Yes	264 (20.1)	193 (19.2)	71 (22.9)	
Antihypertensives, n (%)				0.36
No	871 (66.2)	659 (65.6)	212 (68.4)	
Yes	444 (33.8)	346 (34.4)	98 (31.6)	
Antidiabetics, n (%)				0.193
No	1203 (91.5)	925 (92)	278 (89.7)	
Yes	112 (8.5)	80 (8)	32 (10.3)	
Lipid‐lowering drug, n (%)				0.335
No	928 (70.6)	716 (71.2)	212 (68.4)	
Yes	387 (29.4)	289 (28.8)	98 (31.6)	
NSAIDs, n (%)				<0.001
No	1269 (96.5)	981 (97.6)	288 (92.9)	
Yes	46 (3.5)	24 (2.4)	22 (7.1)	

*Note*: De‐Ritis ratio, the ratio of aspartate aminotransferase (AST) to alanine aminotransferase (ALT); *n*, number; age onset, age of PD onset; onset years, duration from PD onset; race other, includes multiracial; caudate measurement, the uptake value of caudate nucleus on dopamine transporter scan; putamen measurement, the uptake value of putamen on dopamine transporter scan; striatum measurement, the uptake value of striatum on dopamine transporter scan. Numbers that do not add up to 100% are attributable to missing data.

Abbreviations: ALT, alanine aminotransferase; AST, aspartate aminotransferase; BMI, body mass index; ESS, Epworth Sleepiness Scale score; GDS, Geriatric Depression Scale score; MoCA, Montreal Cognitive Assessment; NSAIDs, nonsteroidal anti‐inflammatory drugs; RBC, red blood cell; RBD, REM Sleep Behavior Disorder Questionnaire Score; UPDRS, movement disorder society‐unified Parkinson's disease rating scale; WBC, white blood cell.

Significant differences were observed between the sporadic and familial PD groups in terms of age, sex, years of education, age at onset of PD, disease duration, total protein, serum uric acid, creatinine, MOCA score, GDS score, MDS‐UPDRS Part 1, MDS‐UPDRS Part 2, total MDS‐UPDRS score, and DaTscan measurements (caudate, putamen, striatum). In contrast, no significant differences were found between the two groups regarding BMI, race, total bilirubin, albumin, serum glucose, AST, ALT, ESS score, RBD score, and MDS‐UPDRS Part 3.

### 3.2 Multivariable Logistic Regression Analysis

The results of the multivariable logistic regression analysis are presented in Table 2. The analysis revealed a positive correlation between the AST/ALT ratio and familial PD compared to sporadic PD, with an OR of 1.69 (95% CI: 1.19–2.39, *p* = 0.003) after adjusting for potential confounders, including age, sex, BMI, race, disease duration, UPDRS total score, serum glucose, serum uric acid, albumin, total bilirubin, creatinine, antihypertensives, antidiabetics, lipid‐lowering drug, and NSAIDs. However, no significant association was found between AST or ALT individually and PD genetic subtypes (*p* > 0.05).

**TABLE 2:** Multivariate regression analysis of the association between De‐Ritis ratio and genetic subtypes of Parkinson's disease (PD).

		Model 1		Model 2		Model 3		Model 4	
Variable	*N* total	OR (95% CI)	*p* value	OR (95% CI)	*p* value	OR (95% CI)	*p* value	OR (95% CI)	*p* value
De‐Ritis ratio	1315	1.97 (1.47–2.64)	<0.001	1.64 (1.18–2.29)	0.004	1.59 (1.13–2.23)	0.008	1.69 (1.19–2.39)	0.003
AST	1315	0.98 (0.96–1.00)	0.077	0.99 (0.97–1.01)	0.17	0.99 (0.97–1.01)	0.204	0.98 (0.96–1.01)	0.142
ALT	1315	0.99 (0.98–1.00)	0.054	0.99 (0.98–1)	0.173	0.99 (0.98–1)	0.241	0.99 (0.98–1)	0.135

*Note*: De‐Ritis ratio, the ratio of aspartate aminotransferase (AST) to alanine aminotransferase (ALT); disease duration, duration from PD onset; *n*, number. Model 1: unadjusted. Model 2: adjusted for age, sex, BMI, race, disease duration, and UPDRS total score. Model 3: adjusted for Model 2, serum glucose, serum uric acid, albumin, total bilirubin, and creatinine. Model 4: adjusted for Model 3, antihypertensives, antidiabetics, lipid‐lowering drug, and NSAIDs.

Abbreviations: BMI, body mass index; CI, confidence interval; NSAIDs, nonsteroidal anti‐inflammatory drugs; OR, odds ratio.

### 3.3 Nonlinear Relationship and Inflection Point Analysis

**FIGURE 2:**
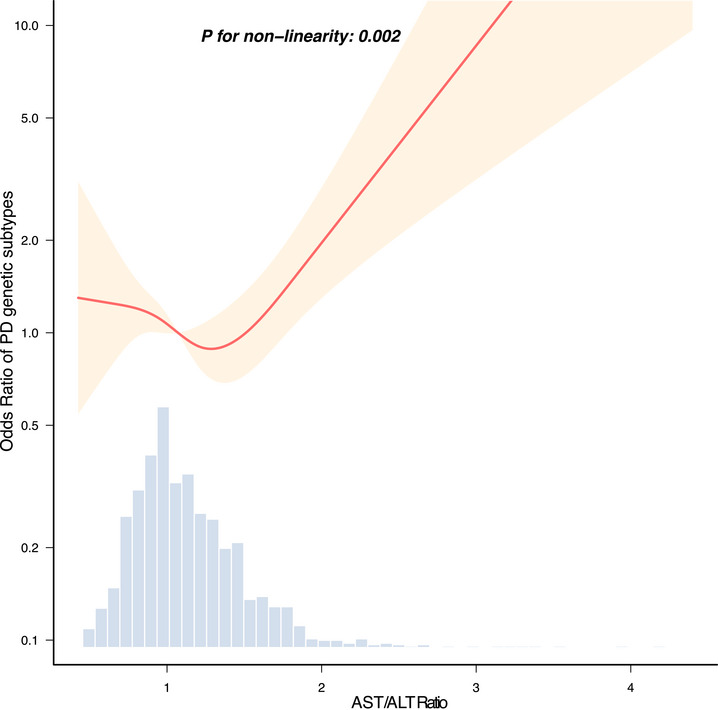
Linear dose–response relationship between AST/ALT ratio and genetic subtypes of PD. Adjustment factors included age, sex, BMI, race, disease duration, UPDRS total score, serum glucose, serum uric acid, albumin, total bilirubin, creatinine, antihypertensives, antidiabetics, lipid‐lowering drug, and NSAIDs. The red line and red area represent the estimated values and their corresponding 95% confidence intervals, respectively. ALT, alanine aminotransferase; AST, aspartate aminotransferase; PD, Parkinson's disease.

The nonlinear relationship analysis, as depicted in Figure 2, demonstrated a U‐shaped relationship between the AST/ALT ratio and familial PD compared to sporadic PD (*p* for nonlinearity = 0.002). The inflection point analysis, detailed in Table 3, identified an inflection point at an AST/ALT ratio of 1.503. Specifically, for AST/ALT ratios below 1.503, the OR was 0.567 (95% CI: 0.286–1.129, *p* = 0.1066), indicating no significant association. In contrast, for AST/ALT ratios greater than or equal to 1.503, the OR was 22.27 (95% CI: 3.726–133.084, *p* = 0.0007), indicating a strong positive correlation.

**TABLE 3:** Threshold effect analysis of De‐Ritis ratio on genetic subtypes of Parkinson's disease (PD).

Threshold of De‐Ritis ratio	OR	95% CI	*p* value
<1.503	0.569	0.286–1.129	0.1066
≥1.503	22.27	3.726–133.084	0.0007

*Note*: Adjustment factors include age, sex, BMI, race, disease duration, UPDRS total score, serum glucose, serum uric acid, albumin, total bilirubin, creatinine, antihypertensives, antidiabetics, lipid‐lowering drug, and NSAIDs.

Abbreviations: CI, confidence interval; OR, odds ratio.

### 3.4 Subgroup Analysis

**FIGURE 3:**
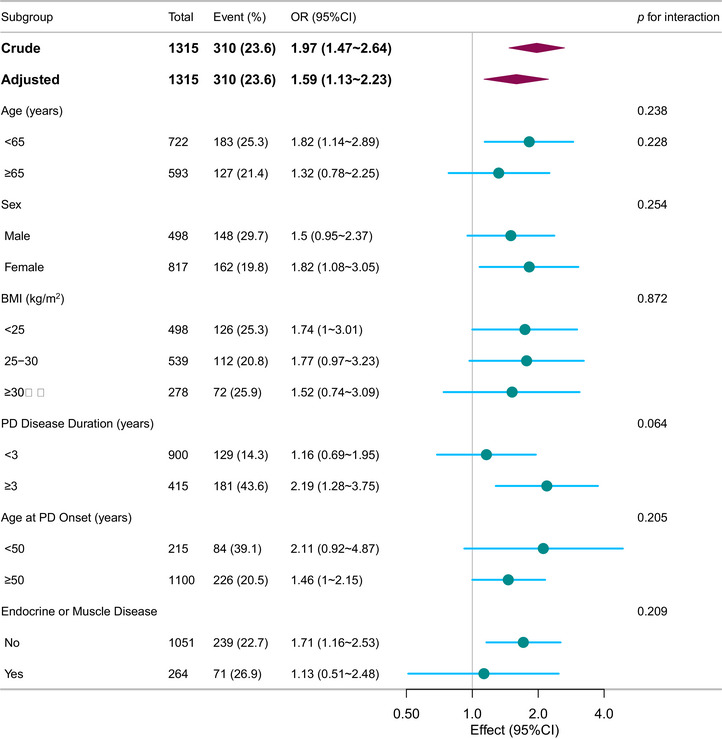
Subgroup analyses of the associations between AST/ALT ratio and genetic subtypes of PD adjusted for age, sex, BMI, race, disease duration, UPDRS total score, serum glucose, serum uric acid, albumin, total bilirubin, creatinine, antihypertensives, antidiabetics, lipid‐lowering drug, and NSAIDs. In each case, the model was not adjusted for the stratification variable. CI, confidence interval; OR, odds ratio; PD, Parkinson's disease.

Subgroup analyses, illustrated in Figure 3, were conducted across various demographic and clinical strata, including age, sex, BMI, age at onset, disease duration, and the presence of any endocrine or muscle disease. The results were consistent across all subgroups, with no significant interaction effects observed.

### 3.5 Sensitivity Analysis

As shown in Table 4, sensitivity analyses confirmed the robustness of the findings. First, after excluding participants with missing data (*N* = 1219), the association remained statistically significant (OR = 1.75, 95% CI: 1.2–2.56, *p* = 0.004). Second, after further excluding participants with abnormal liver function (AST or ALT exceeding the upper limit of normal, *N* = 1149), the association persisted with a slightly higher magnitude (OR = 1.88, 95% CI: 1.26–2.8, *p* = 0.002). Third, after additionally excluding participants diagnosed with any endocrine or muscle disease (*N* = 1051), the association remained robust and consistent (OR = 1.76, 95% CI: 1.19–2.6, *p* = 0.005).

**TABLE 4:** Sensitivity analysis of the association between aspartate aminotransferase (AST)/alanine aminotransferase (ALT) ratio and genetic subtypes of Parkinson's disease (PD).

		Model 1		Model 2		Model 3		Model 4	
Variable	*N* total	OR (95% CI)	*p* value	OR (95% CI)	*p* value	OR (95% CI)	*p* value	OR (95% CI)	*p* value
Exclude patients with missing data	1219	2.13 (1.54–2.94)	<0.001	1.71 (1.18–2.48)	0.004	1.66 (1.14–2.42)	0.008	1.75 (1.2–2.56)	0.004
Exclude patients with abnormal liver function	1149	2.43 (1.74–3.38)	<0.001	1.89 (1.29–2.78)	0.001	1.76 (1.19–2.6)	0.005	1.88 (1.26–2.8)	0.002
Exclude patients with endocrine or muscle disease	1051	2.11 (1.51–2.96)	<0.001	1.77 (1.21–2.59)	0.003	1.71 (1.16–2.53)	0.007	1.76 (1.19–2.6)	0.005

*Note*: De‐Ritis ratio, the ratio of aspartate aminotransferase (AST) to alanine aminotransferase (ALT); onset years, duration from PD onset; *n*, number. Model 1: unadjusted. Model 2: adjusted for age, sex, BMI, race, disease duration, and UPDRS total score. Model 3: adjusted for Model 2, serum glucose, serum uric acid, albumin, total bilirubin, and creatinine. Model 4: adjusted for Model 3, antihypertensives, antidiabetics, lipid‐lowering drug, and NSAIDs.

Abbreviations: BMI, body mass index; CI, confidence interval; OR, odds ratio.

### 3.6 Predictive Model and ROC Analysis

To identify the most significant predictors for differentiating familial PD from sporadic PD, we employed a stepwise selection method based on the adjusted *R*‐squared value. The stepwise selection process resulted in the retention of the following variables in the final model: age, sex, disease duration, AST/ALT ratio, race, albumin, serum glucose, antidiabetics, lipid‐lowering drug, and NSAIDs (Figure S1). Using the selected variables, we constructed a predictive model to evaluate its discriminatory power in differentiating familial PD from sporadic PD. The area under the ROC curve (AUC) for the predictive model was 0.758, with a 95% CI ranging from 0.726 to 0.790 (Figure S2). This AUC value indicates that the model has moderate sensitivity and specificity in distinguishing familial PD from sporadic PD.

## 4 Discussion

To the best of our knowledge, this study represents the first large‐scale retrospective analysis to investigate the relationship between the AST/ALT ratio and the genetic subtypes of PD, specifically focusing on sporadic and familial PD. Our findings revealed a significant association between the AST/ALT ratio and familial PD compared to sporadic PD, with an OR of 1.69 (95% CI: 1.19–2.39, *p* = 0.003) after adjusting for potential confounders. Notably, the AST/ALT ratio showed a nonlinear association with PD genetic subtypes, characterized by an inflection point at 1.503. Below this value, no significant association was observed; above it, the ratio was significantly associated with an increased likelihood of familial PD. Furthermore, both subgroup and sensitivity analyses yielded consistent results, indicating that our conclusions are robust across different subgroups. These findings carry significant clinical implications for the understanding and management of PD risk, potentially offering a novel biomarker for early detection and stratification of PD patients based on their genetic subtype.

Our findings align with and expand upon existing literature regarding the AST/ALT ratio's role in neurodegenerative diseases. Prior studies have demonstrated a negative correlation between the AST/ALT ratio and grip strength and gait speed (Wu et al. 2023; Maeda et al. 2021), both of which are associated with PD (Zheng et al. 2023). Additionally, longitudinal cohort studies have identified the AST/ALT ratio as a biomarker predictive of future PD development, with a hazard ratio of 1.14 (95% CI: 1.08–1.21, *p* = 8.53 × 10^−6^) (Gao et al. 2025). These results underscore the AST/ALT ratio's potential as a valuable biomarker for understanding the broader physiological implications associated with PD. Furthermore, our study extends these findings by revealing a U‐shaped relationship between the AST/ALT ratio and familial PD risk, with an inflection point at an AST/ALT ratio of 1.474. This U‐shaped relationship suggests that both low and high levels of the AST/ALT ratio may be associated with an increased risk of PD, highlighting the importance of maintaining an optimal balance. This finding adds a new dimension to the understanding of the AST/ALT ratio's role in PD, particularly in the context of genetic subtypes.

The AST/ALT ratio is a well‐established marker for liver dysfunction and disease severity (Liu et al. 2018). Notably, it is also associated with nonalcoholic fatty liver disease (NAFLD), which is strongly linked to insulin resistance (Meex and Watt 2017; Khan et al. 2019). Insulin resistance, in turn, has been implicated in PD pathogenesis through multiple convergent mechanisms (Athauda and Foltynie 2016; Sánchez‐Gómez et al. 2020). AST and ALT play crucial roles in the production of glutamate, the primary excitatory neurotransmitter in the central nervous system, which is involved in numerous key brain functions, including synaptic transmission, learning, and memory (Francis 2003). In healthy individuals, plasma levels of ALT and AST are significantly positively correlated with plasma glutamate levels (Kamada et al. 2016), and peripheral blood glutamate levels correlate positively with cerebrospinal fluid glutamate levels (Alfredsson et al. 1988). This suggests that alterations in AST/ALT levels can influence central glutamate availability. In the context of PD, glutamate excitotoxicity has been implicated in disease pathogenesis (Chassain et al. 2008; Gibson et al. 2018). Excessive glutamate synthesis or release, or reduced glutamate reuptake, leads to high glutamate concentrations in the synaptic cleft, causing toxicity and neuronal death. Given the role of AST and ALT in glutamate production, changes in the AST/ALT ratio could potentially impact glutamate homeostasis, thereby influencing neuronal function and contributing to PD pathogenesis.

In Alzheimer's disease, the AST/ALT ratio has been shown to correlate with neurofilament light chain (NfL) levels (Zhang et al. 2024), a biomarker associated with axonal damage and neurodegeneration. Similarly, elevated NfL levels have been proposed as a biomarker for disease severity and progression in PD (Buhmann et al. 2023). The correlation between the AST/ALT ratio and NfL suggests that alterations in AST/ALT levels may reflect underlying neurodegenerative processes, including axonal damage and neuronal.

Brain‐derived neurotrophic factor (BDNF) is a key neurotrophic factor that promotes neuronal survival, differentiation, and synaptic plasticity (Carniel and da Rocha 2021). Lower serum and brain BDNF levels have been reported in PD patients (Scalzo et al. 2010; Howells et al. 2000), leading to increased α‐synuclein expression and impaired dopamine synthesis (Kang et al. 2017). These changes are associated with increased dopaminergic neuronal degeneration, resulting in motor impairments (Baquet et al. 2005) and cognitive deficits (Wang et al. 2016). The positive correlation between serum BDNF levels and the AST/ALT ratio (Yokokawa et al. 2022) suggests that alterations in the AST/ALT ratio may impact BDNF availability, thereby influencing neuroprotective mechanisms and disease progression in PD.

In summary, the AST/ALT ratio's association with PD genetic subtypes may be driven by its impact on glutamate metabolism, neurodegenerative processes, and neurotrophic factor availability. However, the specific mechanisms underlying these associations require further investigation.

This study capitalizes on the extensive and comprehensive data from the PPMI database, encompassing a large cohort of PD patients with detailed clinical, genetic, and biochemical assessments. This robust dataset enhances the robustness and generalizability of our findings. Utilizing multivariate logistic regression models and restricted cubic splines analysis allows for a nuanced understanding of the relationship between the AST/ALT ratio and PD genetic subtypes. The identification of a U‐shaped relationship provides valuable insights into the complexity of this association. Subgroup analyses across various demographic and clinical strata, along with sensitivity analyses, confirm the robustness of our results. The stepwise selection process identified age, sex, age at onset, AST/ALT ratio, race, and albumin as the most significant predictors for differentiating familial PD from sporadic PD. The predictive model, based on these variables, demonstrated moderate discriminatory power with an AUC of 0.758. Our findings suggest that the AST/ALT ratio may serve as a novel biomarker for differentiating genetic subtypes of PD, with significant clinical implications for early detection, stratification, and management. The results of the ROC analysis highlight the need for additional biomarkers and clinical features to enhance the discriminatory power of the model. Future work should focus on validating these findings in larger, independent cohorts and exploring other potential markers that could improve the accuracy of PD subtyping.

Several limitations should be acknowledged. First, the cross‐sectional design prevents causal interpretation, and longitudinal studies are necessary to explore temporal relationships with PD progression. Second, the predominantly European ancestry of the study population (White: 1236, 94.5%) may limit generalizability to other racial or ethnic groups, highlighting the need for validation in more diverse cohorts. Third, although the AST/ALT ratio is a well‐established marker for liver function, its specificity for PD and other neurodegenerative diseases requires further investigation. Lastly, although we adjusted for several potential confounders, residual confounding cannot be entirely ruled out; future studies should consider additional variables that may influence the AST/ALT ratio and PD.

## 5 Conclusions

Our study demonstrates a significant association between the AST/ALT ratio and PD genetic subtypes, with a U‐shaped relationship. The AST/ALT ratio shows potential as a biomarker for early detection and stratification of PD patients. However, further longitudinal and mechanistic studies, especially in diverse populations, are needed to validate these findings and elucidate the underlying mechanisms.

## Author Contributions

**Wen Zhou**: project administration, conceptualization, data curation, supervision, methodology, funding acquisition, writing – original draft, writing – review and editing. **Tianfang Zeng**: supervision, investigation. **Duan Liu**: supervision, software. **Ruijuan Pang**: data curation, visualization.

## Funding

The authors have nothing to report.

## Ethics Statement

This study involved human participants and was approved by the Institutional Review Board of the Michael J. Fox Foundation for Parkinson's Research.

## Consent

All procedures were conducted in accordance with local legislation and institutional requirements, and written informed consent was obtained from all participants.

## Conflicts of Interest

The authors declare no conflicts of interest.

## Supporting information



Figure S1 Stepwise selection process for predictor variables.

Figure S2 ROC curve for the predictive model.

**Supplementary Information**: brb371416‐sup‐0003‐FigureS3.pdf

## Data Availability

The data analyzed in this study are publicly available from Parkinson's Progression Markers Initiative (PPMI) at http://www.ppmi‐info.org.

## References

[brb371416-bib-0001] Alfredsson, G. , F. A. Wiesel , and A. Tylec . 1988. “Relationships Between Glutamate and Monoamine Metabolites in Cerebrospinal Fluid and Serum in Healthy Volunteers.” Biological Psychiatry 23: 689–697. 10.1016/0006-3223(88)90052-2. 2453224

[brb371416-bib-0002] Athauda, D. , and T. Foltynie . 2016. “Insulin Resistance and Parkinson's Disease: A New Target for Disease Modification?” Progress in Neurobiology 145–146: 98–120. 10.1016/j.pneurobio.2016.10.001. 27713036

[brb371416-bib-0003] Baquet, Z. C. , P. C. Bickford , and K. R. Jones . 2005. “Brain‐Derived Neurotrophic Factor Is Required for the Establishment of the Proper Number of Dopaminergic Neurons in the Substantia Nigra Pars Compacta.” Journal of Neuroscience 25: 6251–6259. 10.1523/JNEUROSCI.4601-04.2005. 15987955 PMC6725062

[brb371416-bib-0004] Buhmann, C. , T. Magnus , and C.‐U. Choe . 2023. “Blood Neurofilament Light Chain in Parkinson's Disease.” Journal of Neural Transmission (Vienna) 130: 755–762. 10.1007/s00702-023-02632-7. PMC10199845 37067597

[brb371416-bib-0005] Carniel, B. P. , and N. S. da Rocha . 2021. “Brain‐Derived Neurotrophic Factor (BDNF) and Inflammatory Markers: Perspectives for the Management of Depression.” Progress in Neuro‐Psychopharmacology & Biological Psychiatry 108: 110151. 10.1016/j.pnpbp.2020.110151. 33096156

[brb371416-bib-0006] Chassain, C. , G. Bielicki , E. Durand , et al. 2008. “Metabolic Changes Detected by Proton Magnetic Resonance Spectroscopy In Vivo and In Vitro in a Murin Model of Parkinson's Disease, the MPTP‐Intoxicated Mouse.” Journal of Neurochemistry 105: 874–882. 10.1111/j.1471-4159.2007.05185.x. 18088356

[brb371416-bib-0007] Francis, P. T. 2003. “Glutamatergic Systems in Alzheimer's Disease.” International Journal of Geriatric Psychiatry 18: S15–S21. 10.1002/gps.934. 12973746

[brb371416-bib-0008] Gao, S. , Z. Wang , Y. Huang , et al. 2025. “Early Detection of Parkinson's Disease Through Multiplex Blood and Urine Biomarkers Prior to Clinical Diagnosis.” Npj Parkinson's Disease 11: 35. 10.1038/s41531-025-00888-2. PMC11850829 39994191

[brb371416-bib-0009] GBD 2015 Neurological Disorders Collaborator Group . 2017. “Global, Regional, and National Burden of Neurological Disorders During 1990–2015: A Systematic Analysis for the Global Burden of Disease Study 2015.” Lancet Neurology 16: 877–897. 10.1016/S1474-4422(17)30299-5. 28931491 PMC5641502

[brb371416-bib-0010] Gibson, C. L. , J. T. Balbona , A. Niedzwiecki , et al. 2018. “Glial Loss of the Metallo β‐Lactamase Domain Containing Protein, SWIP‐10, Induces Age‐ and Glutamate‐Signaling Dependent, Dopamine Neuron Degeneration.” PLoS Genetics 14: e1007269. 10.1371/journal.pgen.1007269. 29590100 PMC5891035

[brb371416-bib-0011] Howells, D. W. , M. J. Porritt , J. Y. Wong , et al. 2000. “Reduced BDNF mRNA Expression in the Parkinson's Disease Substantia Nigra.” Experimental Neurology 166: 127–135. 10.1006/exnr.2000.7483. 11031089

[brb371416-bib-0012] Kamada, Y. , R. Hashimoto , H. Yamamori , et al. 2016. “Impact of Plasma Transaminase Levels on the Peripheral Blood Glutamate Levels and Memory Functions in Healthy Subjects.” BBA Clinical 5: 101–107. 10.1016/j.bbacli.2016.02.004. 27051595 PMC4802405

[brb371416-bib-0013] Kang, S. S. , Z. Zhang , X. Liu , et al. 2017. “TrkB Neurotrophic Activities Are Blocked by α‐Synuclein, Triggering Dopaminergic Cell Death in Parkinson's Disease.” PNAS 114: 10773–10778. 10.1073/pnas.1713969114. 28923922 PMC5635931

[brb371416-bib-0014] Khan, R. S. , F. Bril , K. Cusi , and P. N. Newsome . 2019. “Modulation of Insulin Resistance in Nonalcoholic Fatty Liver Disease.” Hepatology 70: 711–724. 10.1002/hep.30429. 30556145

[brb371416-bib-0015] Li, W. , L. Yue , L. Sun , and S. Xiao . 2022. “An Increased Aspartate to Alanine Aminotransferase Ratio Is Associated With a Higher Risk of Cognitive Impairment.” Frontiers in Medicine (Lausanne) 9: 780174. 10.3389/fmed.2022.780174. PMC9021637 35463002

[brb371416-bib-0016] Liu, Y. , P. Zhao , M. Cheng , et al. 2018. “AST to ALT Ratio and Arterial Stiffness in Non‐Fatty Liver Japanese Population: A Secondary Analysis Based on a Cross‐Sectional Study.” Lipids in Health and Disease 17: 275. 10.1186/s12944-018-0920-4. 30509277 PMC6278163

[brb371416-bib-0017] Maeda, D. , N. Kagiyama , K. Jujo , et al. 2021. “Aspartate Aminotransferase to Alanine Aminotransferase Ratio Is Associated With Frailty and Mortality in Older Patients With Heart Failure.” Scientific Reports 11: 11957. 10.1038/s41598-021-91368-z. 34099767 PMC8184951

[brb371416-bib-0018] Marek, K. , D. Jennings , S. Lasch , et al. 2011. “The Parkinson Progression Marker Initiative (PPMI).” Progress in Neurobiology 95: 629–635. 10.1016/j.pneurobio.2011.09.005. 21930184 PMC9014725

[brb371416-bib-0019] Meex, R. C. R. , and M. J. Watt . 2017. “Hepatokines: Linking Nonalcoholic Fatty Liver Disease and Insulin Resistance.” Nature Reviews Endocrinology 13: 509–520. 10.1038/nrendo.2017.56. 28621339

[brb371416-bib-0020] Nho, K. , A. Kueider‐Paisley , S. Ahmad , et al. 2019. “Association of Altered Liver Enzymes With Alzheimer Disease Diagnosis, Cognition, Neuroimaging Measures, and Cerebrospinal Fluid Biomarkers.” JAMA Network Open 2: e197978. 10.1001/jamanetworkopen.2019.7978. 31365104 PMC6669786

[brb371416-bib-0021] Poewe, W. , K. Seppi , K. Marini , and P. Mahlknecht . 2020. “New Hopes for Disease Modification in Parkinson's Disease.” Neuropharmacology 171: 108085. 10.1016/j.neuropharm.2020.108085. 32298705

[brb371416-bib-0022] Sánchez‐Gómez, A. , G. Alcarraz‐Vizán , M. Fernández , et al. 2020. “Peripheral Insulin and Amylin Levels in Parkinson's Disease.” Parkinsonism & Related Disorders 79: 91–96. 10.1016/j.parkreldis.2020.08.018. 32911247

[brb371416-bib-0023] Scalzo, P. , A. Kümmer , T. L. Bretas , F. Cardoso , and A. L. Teixeira . 2010. “Serum Levels of Brain‐Derived Neurotrophic Factor Correlate With Motor Impairment in Parkinson's Disease.” Journal of Neurology 257: 540–545. 10.1007/s00415-009-5357-2. 19847468

[brb371416-bib-0024] Simon, D. K. , C. M. Tanner , and P. Brundin . 2020. “Parkinson Disease Epidemiology, Pathology, Genetics, and Pathophysiology.” Clinics in Geriatric Medicine 36: 1–12. 10.1016/j.cger.2019.08.002. 31733690 PMC6905381

[brb371416-bib-0025] Sulzer, D. 2007. “Multiple Hit Hypotheses for Dopamine Neuron Loss in Parkinson's Disease.” Trends in Neuroscience (Tins) 30: 244–250. 10.1016/j.tins.2007.03.009. 17418429

[brb371416-bib-0026] Tolosa, E. , M. Vila , C. Klein , and O. Rascol . 2020. “LRRK2 in Parkinson Disease: Challenges of Clinical Trials.” Nature Reviews Neurology 16: 97–107. 10.1038/s41582-019-0301-2. 31980808

[brb371416-bib-0027] Wang, Y. , H. Liu , B.‐S. Zhang , J. C. Soares , and X. Y. Zhang . 2016. “Low BDNF Is Associated With Cognitive Impairments in Patients With Parkinson's Disease.” Parkinsonism & Related Disorders 29: 66–71. 10.1016/j.parkreldis.2016.05.023. 27245919

[brb371416-bib-0028] Wu, C. , Q. Wang , C.‐Y. Zhou , et al. 2023. “Association of AST/ALT (De Ritis) Ratio With Sarcopenia in a Chinese Population of Community‐Dwelling Elderly.” Heliyon 9: e20427. 10.1016/j.heliyon.2023.e20427. 37822616 PMC10562753

[brb371416-bib-0029] Yokokawa, T. , S. Sasaki , K. Sase , et al. 2022. “Association of Serum Brain‐Derived Neurotrophic Factor With Hepatic Enzymes, AST/ALT Ratio, and FIB‐4 Index in Middle‐Aged and Older Women.” PLoS ONE 17: e0273056. 10.1371/journal.pone.0273056. 35998179 PMC9398011

[brb371416-bib-0030] Zhang, B. , C. Zhang , Y. Wang , et al. 2024. “Alzheimer's Disease Neuroimaging Initiative. Associations of Liver Function With Plasma Biomarkers for Alzheimer's Disease.” Neurological Sciences 45: 2625–2631. 10.1007/s10072-023-07284-9. 38177970

[brb371416-bib-0031] Zheng, Z. , Y. Lv , S. Rong , T. Sun , and L. Chen . 2023. “Physical Frailty, Genetic Predisposition, and Incident Parkinson Disease.” JAMA Neurology 80: 455–461. 10.1001/jamaneurol.2023.0183. 36912851 PMC10012040

[brb371416-bib-0032] Zholdasbekova, G. , A. Kaiyrlykyzy , A. Kassenova , D. Alzhanova , D. Klyuev , and S. Askarova . 2024. “ApoE Gene Polymorphism and Clinical, Biochemical, and Sociodemographic Characteristics of Alzheimer's Disease Patients From Northern and Southern Regions of Kazakhstan.” International Journal of Geriatric Psychiatry 39: e70019. 10.1002/gps.70019. 39568323 PMC11579630

[brb371416-bib-0033] Zhou, W. , T. Zeng , D. Liu , R. Pang , and L. Gong . 2025. “Association Between Inflammatory Markers (SII and SIRI) and Anxiety Levels in Parkinson's Disease.” Frontiers in Psychiatry: 16. 10.3389/fpsyt.2025.1635817. PMC12436417 40964431

