# Supplementary figures and images for: ASL/ALT Ratio in Familial and Sporadic Parkinson's Disease: Insights From Cross‐Sectional Logistic Analysis
Source: Brain Behav. 2026 Apr 22;16(4):e71416. doi: 10.1002/brb3.71416 (PMC13103267; doi:10.1002/brb3.71416)

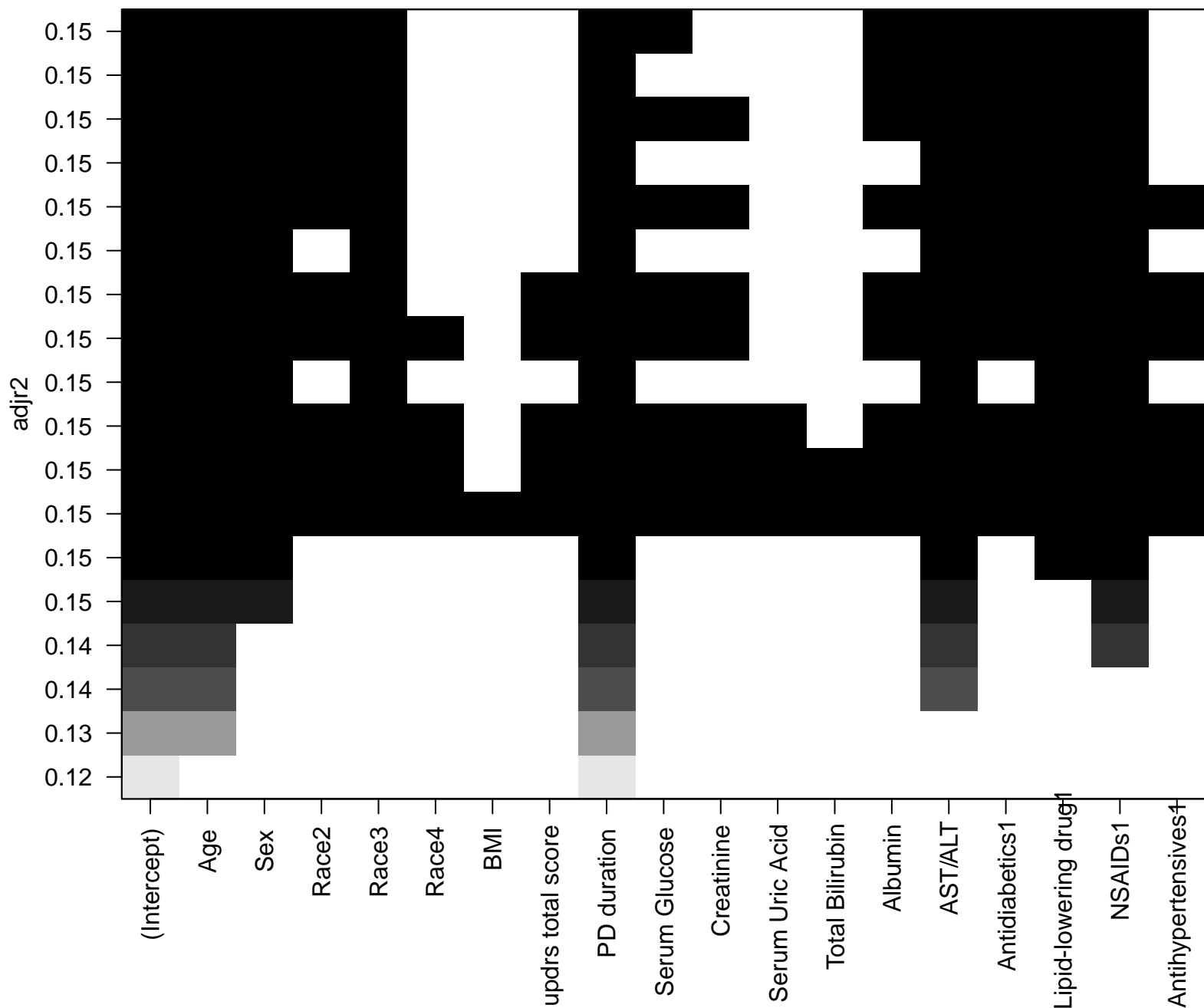

Supplement: Supplementary file 1 — Figure S1 Stepwise selection process for predictor variables. [file BRB3-16-e71416-s001.pdf]

Model prediction

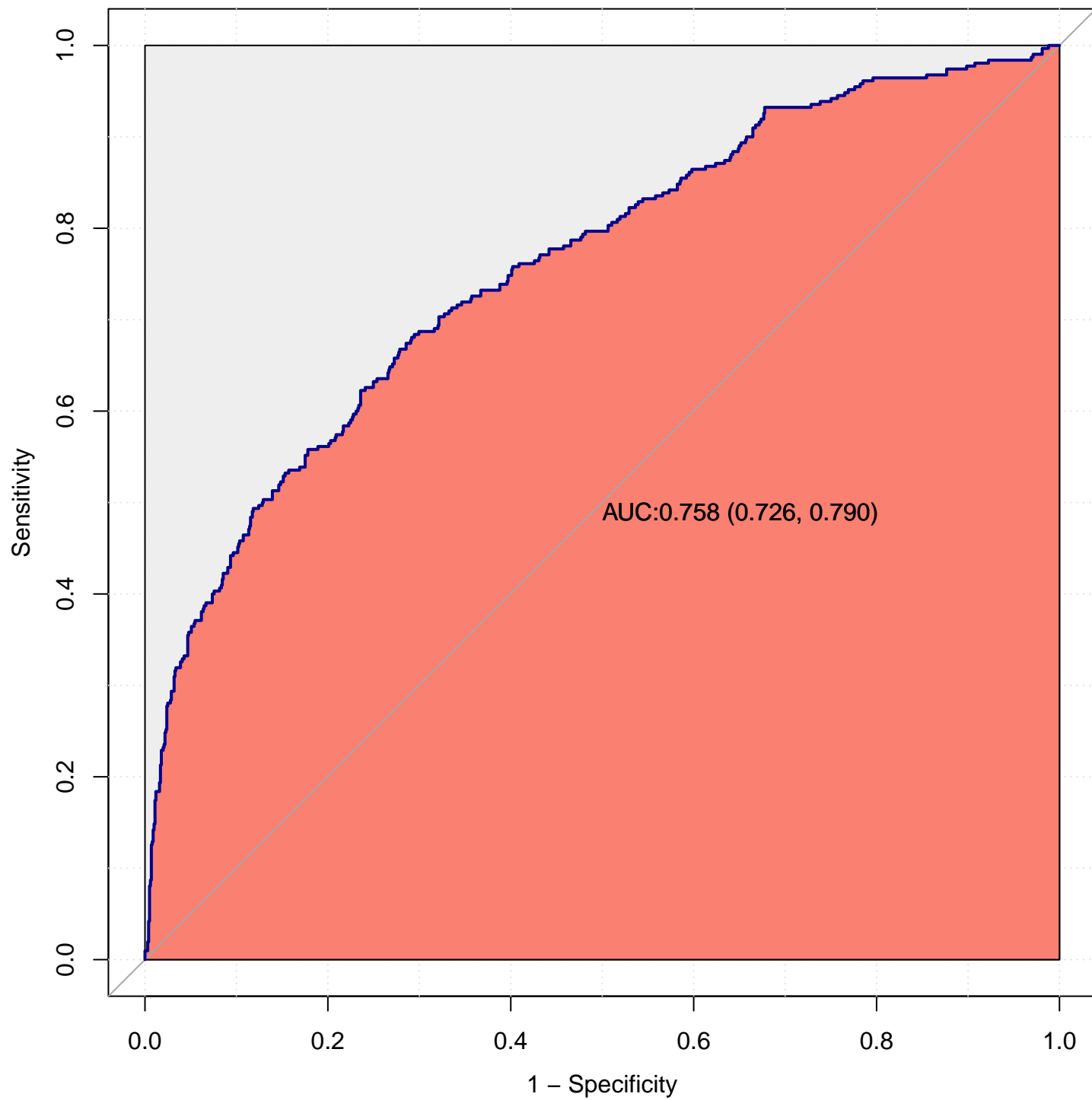

Supplement: Supplementary file 2 — Figure S2 ROC curve for the predictive model. [file BRB3-16-e71416-s003.pdf]
