# Supplementary material for: ASL/ALT Ratio in Familial and Sporadic Parkinson's Disease: Insights From Cross‐Sectional Logistic Analysis
Source: Brain Behav. 2026 Apr 22;16(4):e71416. doi: 10.1002/brb3.71416 (PMC13103267; doi:10.1002/brb3.71416)
Supplement: Supplementary file 3 — Supplementary Information: brb371416‐sup‐0003‐FigureS3.pdf [file BRB3-16-e71416-s002.pdf]

CENTRAL ILLUSTRATION: AST/ALT ratio helps identify familial Parkinson's disease compared to sporadic PD

Multivariable logistic regression revealed a significant positive association between the AST/ALT ratio and familial PD (adjusted OR = 1.69, 95% CI: 1.19–2.39, p = 0.003)

Restricted cubic splines revealed a nonlinear relationship with an inflection point at 1.503; above this threshold, the association was markedly stronger (OR = 22.27, p = 0.0007), with no significant association below.

Study Population

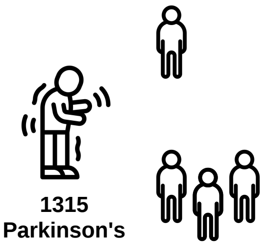

1315 Parkinson's disease

1005 Sporadic PD

310 Familial PD

Research Contents

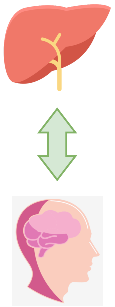

AST/ALT

PD genetic subtypes

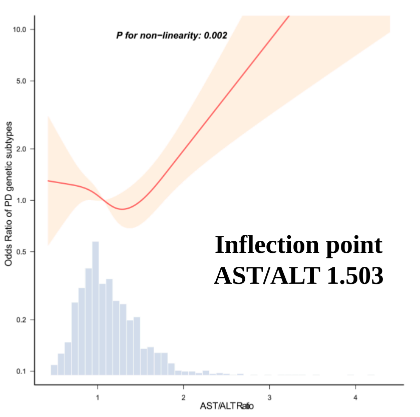

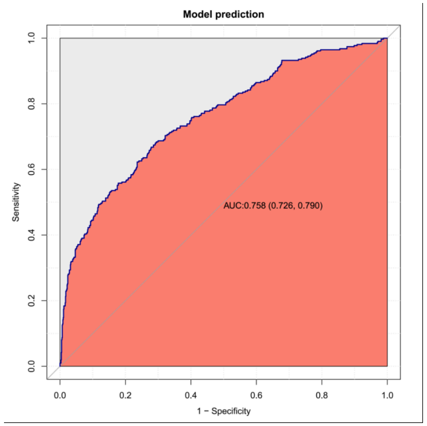

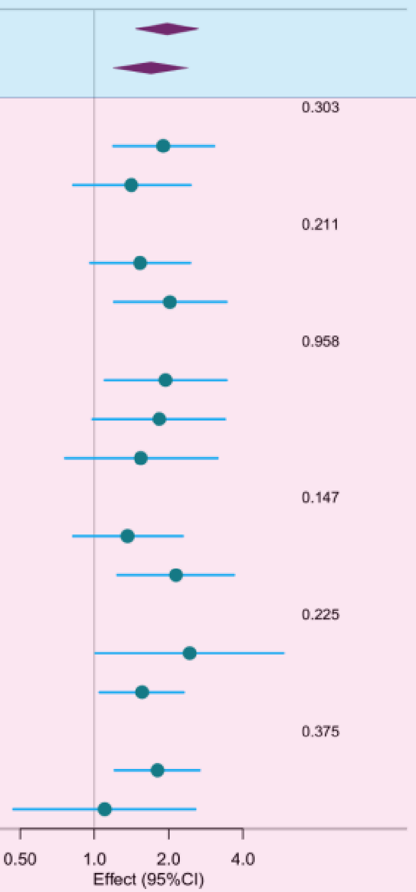

Subgroup	Total	Event (%)	OR (95%CI)	P for interaction
**Crude**	**1315**	**310 (23.6)**	**1.97 (1.47~2.64)**	
**Adjusted**	**1315**	**310 (23.6)**	**1.69 (1.19~2.39)**	
Age (years)				0.303
<65	722	183 (25.3)	1.9 (1.19~3.05)	
≥65	593	127 (21.4)	1.41 (0.82~2.45)	
Sex				0.211
Male	498	148 (29.7)	1.53 (0.96~2.44)	
Female	817	162 (19.8)	2.02 (1.2~3.43)	
BMI (kg/m²)				0.958
<25	496	125 (25.2)	1.94 (1.1~3.43)	
25–30	540	113 (20.9)	1.83 (0.98~3.38)	
≥30	279	72 (25.8)	1.54 (0.76~3.15)	
PD Disease Duration (years)				0.147
<3	899	127 (14.1)	1.36 (0.82~2.28)	
≥3	416	183 (44)	2.14 (1.24~3.67)	
Age at PD Onset (years)				0.225
<50	215	84 (39.1)	2.43 (1.01~5.84)	
≥50	1100	226 (20.5)	1.56 (1.05~2.3)	
Endocrine or Muscle Disease				0.375
No	1051	239 (22.7)	1.8 (1.21~2.67)	
Yes	264	71 (26.9)	1.1 (0.47~2.56)	
